# Wounded Intestines

**Published:** 1837

**Authors:** 


### WESTERN JOURNAL

OF THE

### MEDICAL AND PHYSICAL SCIENCES:

**For January, February, and March, 1837.**

### ESSAYS AND CASES.

### Art. I.—Wounded Intestines.

*History of a case of Wounded Intestines and Omentum, successfully treated.* Extracted from the Inaugural Thesis of Aquila Toland, M. D., of Madison county, Ohio; a graduate of the Medical Department of the Cincinnati College.

On the 25th of August, 1836, I was called to visit W. B., aged twenty, an athletic young man, of a sanguine temperament, who in handling a scythe in a careless manner, with his foot upon the nib or handle and the point turned towards his body, leant forward, and his foot slipt from its position and forcibly brought the point of the instrument agaisnt the abdomen. It pierced the parieties, an inch or so, above and to the left of the umbilicus, in the line of the fibres of the rectus muscle, about midway between the linea alba and the linea semilunaris. There was a longitudinal wound inflicted through the parieties near five inches in length externally—internally the wound was about three inches and an half. The omentum was perforated, and the small intestines suffered four wounds. The first nearly divided the gut transversely, the second cut it rather more than half off in the same direction, the third made a longitudinal fissure of about two inches in length, the fourth was a mere puncture, but yet sufficient for the escape of the contents of the intestine. The accident occurred in a wild meadow about half a mile from the house, and he was hauled home upon a sled, in as easy a manner as possible, an attendant being placed by his side for the purpose of supporting his bowels, which had largely protruded. The distance I had to ride was about eight miles, and when I arrived a large mass of the small intestines, together with a portion of the colon and omentum were found in a pile, covered with blood and their own extravasated contents, which had been very abundantly poured out upon them. Nothing had been done for his relief, save only that his friends had taken the precaution to cover his bowels with some linen cloths, to keep them warm and screen them as they supposed from the effects of the atmosphere. In this situation they had lain out near four hours. The omentum being wounded, had thrown out a considerable quantity of blood, and this as well as their own contents which had also been poured out, had dried upon their peritoneal covering. There was a frequent wiry pulse, constant nausea and vomiting, the surface and extremities were colder than usual, and bathed in a clammy perspiration; in short, all the symptoms of strangulated hernia were present; and, indeed, there *was* strangulation, for the abdominal muscles, from being divided chiefly in the longitudinal direction of of their fibres, had closed upon the protruded mass, more particularly upon the neck of the mesenteric portion of it. From the jejunum which had four perforations, and was at least partially strangulated, there was a constant escape of gaseous and fœcal contents. The lips or edges of the wounds in the intestines, presented an everted appearance. This eversion was produced by the elongation of the mucus membrane, which completely covered the cut edges of the peritoneal and muscular coats; in the longitudinal cut the eversion was less apparent.

The accident occurred soon after dinner, and as he had vomited much, what he had eaten was of course ejected; and in consultation with Dr. Wilson, who assisted me in the operation, we came to the conclusion to make soft gentle pressure upon the bowels, in such manner as thoroughly to empty them of their contents, and thereby moderate the symptoms of strangulation, as well as obviate a cause of aggravation to the subsequent inflammation. We, then, by the application of tepid milk and water, cleansed the surface of the bowels from the dried blood and fœcal substances, and proceeded to bring the lips of the wounds in the intestines together, by suture. This was done, without including the mucous coat. The ligatures used were broad, and composed of very fine flax thread slackly twisted. The lips of the transverse wound, which had so nearly divided the gut, were brought together, and retained in their proper position, by the glover's suture—that is, the ligature was armed at each end, and with a needle of small size, and the points of the needles were carefully entered at each side, on the inner edge of the wound between the mucous and muscular coats, and passed alternately from side to side, the former being pushed back, and not included in the ligature. All the other wounds were brought together by the interrupted suture, the needle being entered as above described.

The next indication, which was to return the bowels into their proper cavity, was now attempted, but found to be impracticable by gentle means, until the wound in the inner parieties which as before observed, was shorter than that externally, was enlarged and this I readily accomplished by a blunt pointed bistory. I have, however, neglected to remark, that the lower and most depending portion of the wound, was abruptly transverse, inclining to the left, in the direction of the superior, anterior, spinous process of the ilium. It was this portion which was enlarged. The edges of the longitudinal wound were then brought into apposition, and retained by the quilled suture. The transverse portion of the wound was left open, with a view to permit the escape of any extravasated matter, which might already exist in the peritoneal cavity, be produced by effusion or suppuration.

Immediately upon the return of the bowels, the patient expressed himself relieved. The nausea and vomiting, which had been so urgent, and which were greatly aggravated during the operation, now abated; there was an increase in the volume of the pulse, and its frequency was diminished. The surface of the body, which had been bathed in a profuse cold clammy perspiration, now appeared gradually to regain its activity and his extremities became warm. Previous to the commencement of the operation, opium both in the solid and liquid form, had been administered, but had been speedily rejected; one grain and an half of pulverized opium was now given and retained, which appeared to have a tranquilizing effect. Mucilaginous drinks composed of the recent bark of the elm (Ulmus Americana) in cold water, were directed for his common drink, and cold injections of the same substance were ordered to be thrown into the rectum every three hours. It was now about sun down, and I left him for the night.

August 26th. This morning about 10 o'clock, I visited him. He had passed a bad night; experienced several severe chills, and now had a violent fever; his pulse was frequent and rather tense, the tongue dry, red at the tip and red round the edges, with a streak of brown fur along the middle; the skin was dry, the urine rather scanty and high colored.

He complained of some headache, had drunk largely of the cold elm bark water, but was still thirsty, and there was an uneasy sense of tension and fullness, in the abdomen. I immediately opened a vein, and drew about twenty ounces of blood in a full stream, which produced a decided impression upon the pulse, attended with slight syncope, and was followed by a copious perspiration. I gave him no medicine, but directed the mucilage to be continued for his common drink, cold injections of the same to be administered every three hours; and left particular directions, that he be allowed no other nourishment during my absence.

27th, 10 o'clock. He had been more comfortable, obtained some refreshing sleep through the night, there was not much fever, his tongue was moist, and there was rather less thirst, but he still called, frequently, for the elm water. He remarked, that the cold ejections were in the highest degree refreshing, and desired me to permit them to be used more frequently. There had been no alvine discharge, there was a great fulness and tension in the hypogastriac region, and upon introducing a blunt pointed probe into the transvere wound, which had been left open, I discovered that a portion of omentum had insinuated itself into the wound and closed the aperture. When this was pushed back, a copious discharge of purulent matter mixed with blood issued from the wound, which speedily relaxed the distention, and relieved the oppression. The mucilaginous drink and injections were directed to be continued, and no other nourishment was allowed.

28th. He had been pretty comfortable through the night, had some refreshing sleep and but little fever, his pulse was yet a little too frequent, there had been several copious discharges from the wound; and he now for the first time, expressed a desire for some nourishment, but I directed only a tea spoonful of gum arabic with the same quantity of sugar, dissolved in cold elm water, to be given every three hours by way of diet—the other treatment to be continued.

29th. He was doing well; had but little fever, and the wound still discharged freely, whenever the omentum was pushed back, and this was directed to be done frequently in my absence. I still limited him to the same quantity of gum arabic, and the same quantity of sugar as before, and directed the former treatment to be continued.

30th. Every thing going on well, the wound still discharging freely when opened, and no change made in the treatment.

31st. No fever. There has been a free discharge from his bowels, for the first time since the accident. The dejection was dark and offensive, his appetite was excellent, and I now directed, at his own urgent request, that he be allowed a pint of squirrel broth in broken doses, at regular intervals, in addition to his former allowance, the other treatment as before to be continued.

Sept. 1. He was worse; had slept but little, his pulse was considerably accelerated, and his tongue rather dry, the thirst had increased, and his appetite failed; the wound still continued to] discharge freely, and threw out purulent matter of little consistence, colored with blood. I prohibited the broth, and desired him to confine himself to the gum and sugar as before, the other treatment being continued.

2d. There was an abatement of fever, no thirst, and his appetite had returned, there had been another alvine discharge.

3d. No fever or distention of the abdomen, his appetite fine, and rather hard to control—the treatment and diet continued.

4th. Symptoms all favorable; very hungry; there was but little discharge from the wound, which was now nearly closed by adhesion with the omentum. I now allowed him an ounce of the gum arabic with the same quantity of sugar, and continued the treatment as before directed.

5th. The wound nearly closed; but little discharge and no sense of fullness; all the other symptoms favorable; his appetite was very urgent, and he complained of starvation. I now increased his allowance of gum and sugar, and applying a broad bandage round his abdomen, permitted him to exercise a little, by walking his room when he chose; for he had been confined to the recumbent position ever since the occurrence of the accident.

6th. He had been using some exercise; his only complaint was, the want of food; his bowels were in a good condition. I allowed him as much of the gum and sugar as he chose, and the cold injections were discontinued.

7th. Did not visit him. 8th. Found him quite well, and walking about, but still very hungry; there was no inconvenience whatever experienced in the abdomen, and the rigid abstinence to which he had been subjected for fourteen days, was commuted for a more generous diet of mush and milk.

The wound was now nearly cicatrised; there was no inconvenience experienced in the region where it was inflicted, and his bowels were regular, and my attendance was no longer necessary. Some weeks afterwards I saw him in excellent health, in which condition, as I am informed, he still continues.

This case occurred eight miles east of London, the seat of justice of Madison county, Ohio, in a district of country, which from the nature of its soil and the aspect of its surface, is favorable to the evolution of miasmata. Remittent and intermittent fevers, were prevalent in the neighborhood at the time of the accident. From a mild attack of the latter of which he had recovered only about three weeks previously.

It will be observed, that during the whole course of treatment, which lasted fourteen days, the patient took no medicine that was retained upon his stomach, with the exception of about one grain and a half of opium. The mucilage which was so largely employed, was extracted from the recent inner bark of the slippery elm, by the solvent action of cold water. And here I will remark, that this mucilage is better, and more abundantly extracted, by cold than by warm water, and is far more agreeable to the patient, who frequently prefers it to water alone. In dysentery and gastritis, and in all diseases wherein the mucus membranes are involved, where mucilages are indicated (and they are indicated more frequently than is generally supposed) we have for the mucilage of the slippery elm bark no substitute. I have long since been of opinion, that cold mucilaginous injections are too much neglected.

The most depending portion of the external wound, was kept open, and large quantities of purulent matter mixed with blood escaped. What would have been the effects produced by its retention? It would seem that it could never have been removed by the absorbents, and if suffered to remain and stagnate, the effects must have been disastrous. When effusion takes place into the peritoneal cavity, to any great extent, every mode of treatment heretofore employed, has generally proved unavailing; and yet pathological investigations have shown, that extensive inflammation may exist in the peritoneum without extending to the subjacent structures. Might not the practice of puncturing the abdomen in chronic peritonitis be adopted with advantage? Bayle, Scoulteten, Abercrombie, Pemberton, Armstrong and others, all tell us, that this cavity upon post mortem examination, is found to contain large quantities of purulent matter, mixed with coagula of blood, or fluids of a yellow or milky appearance; that the intestines are often glued together by the intervention of false membranes, that sacs are formed by membranous exudations, and that the intestines present thickened appearance, tubercular granulations and various other morbid productions.

Most writers account for the difficuity of curing this form of peritoneal inflammation, by referring it to the impossibility of producing the absorption of the tubercular matter, which is deposited between the layers of the peritoneum. Now the retention of the fluids is infinitely more dangerous, than would be an operation for their removal, and an opening might be made through the parieties of the abdomen, the intestines examined, their adhesions broken up, and their fluids evacuated, and perhaps those granulations, and tubercular deposits removed, with less handling, and much less exposure to the effects of the atmosphere, and other irritating effects than that which was suffered in the case under consideration. For in this case it will be observed, that there was not only a long wound through the walls of the abdomen, but the omentum and intestines were also extensively wounded, had lain out for at least four hours, with blood and other extravasations dried upon their surface, and that they had to be washed, stitched and handled, suffering all the time with at least partial strangulation, and yet the patient recovered, completely in fourteen days. There was inflammation and effusion certainly to a considerable extent, and at least three or four quarts of matter discharged from the wound. Had this matter been pent up in this peritoneal cavity and suffered to stagnate, will any one believe, that the patient could have recovered? The absorbents could not have removed this large amount of fluid.

By a review of the facts presented in this case, we must, also, arrive at the conclusion, that the operation for the relief of intussusception, is entirely practicable and should be frequently resorted to as a remedy for this dangerous affection. This operation has been recommended and practiced a few times only with success, owing probably to delay in its performance, for perhaps as much promptness would be required here as in the operation for strangulated hernia. Bloodletting, drastic cathartics, clysters of tobacco, quicksilver by the pound, purgatives when the enveloped gut is drawn upwards, and emetics when drawn downwards, have been advised and employed without order or success, and the patient dies. But when we have employed bleeding to its full extent, mild but efficient purgatives, the warm bath and stomach tube to the rectum, and the antispasmodic powers of opium, without success, we have done all, perhaps, that can reasonably be expected from the agency of medicine, and the operation of gastrotomy should be resorted to, without delay. The operation will not probably be more formidable to the patient, or difficult to the operator, than that so frequently resorted to for the relief of strangulated inguinal hernia. Post mortem dissection of patients who have died of this disease, has shown, that the invagination generally exists in the right iliac fossa, near the junction of the small with the large intestines, and this as one of our guides, would seem to indicate, that through the linea semilunaris of that region, would be the point for the performance of the operation.

The diagnosis of ileus though frequently difficult, will be generally found sufficiently clear, to distinguish it from all other diseases, except perhaps some concealed cases of strangulated hernia. Hernial tumors are sometimes found presenting themselves at the foramen ovale, at the ischiatic notch, and through the diaphragm or mesentery. The existence of these tumors, and their situation, have been clearly established by post mortem examinations, and their symptoms during the life of the patient, have been found to be about the same as those of ileus, or strangulations in other parts. The remedies recommended in these cases, have of course amounted to but little, and are frequently worse than nothing, and generally after a period of short but intense suffering, gangrene and death are the result. Should we therefore even fail in our diagnosis and proceed to operate for volvulus, when the disease was one of those concealed cases of hernia, the error would be of but little consequence, for an operation for the relief of the latter, would certainly seem to me to afford as much hope of success, and offer the patient as fair a chance for his life, as any other plan of treatment at present recommended.

I have, therefore, but little doubt, that the period is not distant, when surgeons will proceed with as little hesitation, to the performance of the operation above indicated, as they now do, to operate for strangulated femoral hernia; and the latter strangulation too, when the tumor is small, of but recent occurrence and consisting principally of intestine, might probably, be relieved, with as much facility to the surgeon and more safety to the patient, by the supra pubic operation. The protruded gut might be gently drawn back, and its re-emergence prevented by the immediate application of a truss. The anatomical structure of the parts through which we have to cut being seldom understood, their proximity to the great crural vessels and the parasitic course of the absturator artery, render the usual operation exceedingly difficult to the inexperienced surgeon, and dangerous to the patient on account of hæmorrhage.

*February*, 1837.

### Editorial Note.

We subjoin to Dr. Toland's narrative of this interesting case, an extract from Cooper's First Lines of the Practice of Surgery, on the subject of wounds of the intestines. It contains a digest of various opinions, and the book may not be in the hands of all our insulated readers. It will be seen, that the practice so successfully pursued by Dr. Toland, was rather in opposition to that of the surgeons generally.

"We are next to consider the case, in which the protruded bowel is still more extensively, or even totally divided. Here the admirers of the needle have found ample scope for their ingenuity; and since very few of them have met with cases exactly of this description in the human subject, they have made a variety of experiments on animals, in order to determine the right mode of treatment. Some of these reports are favorable to the practice of sewing up the wounded bowel. Ramdohr is stated to have actually cut off a large part of a mortified intestine in the human subject, and to have joined the sound ends together, by inserting the upper within the lower one, and fixing them in this position with a suture; the ligature being also employed to keep them near the external wound. The patient recovered, and the feces afterwards passed entirely the natural way. About a year after the operation, the patient died, when the anitomical preparation of the parts was sent to Heister. They were preserved in spirit of wine, and exhibited, according to this last author, a union of the two ends of the bowel, and their consolidation with a part of the abdomen. Now, it has been reasonably questioned, whether the union here spoken of, ever really happened. When the upper end of the bowel is introduced into the lower, the external surface of the former is put in contact with the inner one of the latter; a serous membrane is placed in contact with a mucous one. These heterogeneous structures, Richerand alleges, are not disposed to unite. The mucous membrane, when inflamed, more readily secretes a kind of mucus, which must be an invincible obstacle to adhesion. He thinks it therefore more than probable, that, in the case related by Heister, the invagination was maintained by the union of the intestine with the corresponding part of the abdominal parietes. Several experiments on living animals have convinced him that this happens, and that the mucous membrane will not unite with the external peritoneal coat. If this be a fact, it is of course a strong argument against repeating Ramdohr's practice. Another objection is, that the upper end of the bowel cannot be put into the lower one, unless it be separated from a part of the mesentery, and a division of the mesenteric arteries would cause a dangerous bleeding. In vain did Boyer tie seven or eight of these vessels; his patient died with an extravasation in the abdomen. The difficulties, encountered by Moebius and Dr. Smith, in their attempts to repeat this experiment on animals, are related in my dictionary, and I need not, therefore, expatiate upon them. In short, experience is decidedly adverse to Ramdohr's practice, either in its original form, or modified by the ingenious introduction of cylinders of isinglass, pasteboard, etc. Flajani tried the artifice on several patients, under his care, in the hospital at Rome, but death was invariably the consequence. I am of opinion that Mr. Traverse deserves the thanks of the profession, for the attention and talent with which he has investigated the subject before us; but with respect to the question of sutures, I apprehend, that he has gone too far, much too far, when he declares, that, in order to avoid abdominal effusion, the suture employed should be such as will secure the absolute contact of the everted surfaces of the divided intestine.

When the intestine has been completely divided with a cutting instrument, Scarpa is decidedly of opinion, that Ramdohr's operation cannot be undertaken with any probability of success. But, setting out of the question this bold method, at once so amusing and captivating to the inexperienced student, this eminent professor offers a variety of arguments against sewing the intestines at all, and asserts, that, *in all cases of penetrating wounds of the abdomen, attended with injury of the intestine, whether the canal be opened longitudinally, or transversely, a suture is always not merely useless, but even dangerous and fatal.* In whatever manner it is practised, says he, one cannot avoid the evils which must originate from the punctures, however few, and from the passage of the ligatures through the coats of the intestine; a part endued with exquisite sensibility, and whose external tunic is much disposed to inflame, and rapidly to communicate the inflammation to all the other abdominal viscera. It has (says Scarpa) been unfortunately proved, by the experience of several ages, that, in most of the cases, in which the intestine has been stitched in penetrating wounds of the belly, the patients have died in the greatest agony. If a few escaped the dangers of this operation, it was only because in them the stitches soon cut their way out, and were voided with the feces, which continued to escape from the wound until it was entirely healed. * * * * * * *

"The very nature of the process, by which the reparation of wounds of the bowels is effected, is a weighty argument against the employment of a suture. In their cicatrization, they follow quite a different course from that of simple wounds of the skin, muscles, or any other parts of the body. Their edges never become immediately applied to each other, and therefore, strictly speaking, they do not reunite. Their cure is altogether completed through the medium of the surrounding parts; that is to say, by the adhesions which the intestines contract with the great sac of the peritoneum lining the cavity of the abdomen, or with the productions of this membrane, which compose the external covering or the greater part of the viscera."

